# Effects of multi-stakeholder platforms on multi-stakeholder innovation networks: Implications for research for development interventions targeting innovations at scale

**DOI:** 10.1371/journal.pone.0197993

**Published:** 2018-06-05

**Authors:** Murat Sartas, Marc Schut, Frans Hermans, Piet van Asten, Cees Leeuwis

**Affiliations:** 1 Knowledge, Technology and Innovation Group, Wageningen University, Wageningen, The Netherlands; 2 International Institute of Tropical Agriculture (IITA), Kigali, Rwanda; 3 Swedish University of Agricultural Sciences (SLU), Rural development and natural resource management, Uppsala, Sweden; 4 Leibniz Institute for Agricultural Development in Transition Economies (IAMO), Halle (Saale), Germany; 5 International Institute of Tropical Agriculture (IITA), Kampala, Uganda; Pisa University, ITALY

## Abstract

Multi-stakeholder platforms (MSPs) have been playing an increasing role in interventions aiming to generate and scale innovations in agricultural systems. However, the contribution of MSPs in achieving innovations and scaling has been varied, and many factors have been reported to be important for their performance. This paper aims to provide evidence on the contribution of MSPs to innovation and scaling by focusing on three developing country cases in Burundi, Democratic Republic of Congo, and Rwanda. Through social network analysis and logistic models, the paper studies the changes in the characteristics of multi-stakeholder innovation networks targeted by MSPs and identifies factors that play significant roles in triggering these changes. The results demonstrate that MSPs do not necessarily expand and decentralize innovation networks but can lead to contraction and centralization in the initial years of implementation. They show that some of the intended next users of interventions with MSPs–local-level actors–left the innovation networks, whereas the lead organization controlling resource allocation in the MSPs substantially increased its centrality. They also indicate that not all the factors of change in innovation networks are country specific. Initial conditions of innovation networks and funding provided by the MSPs are common factors explaining changes in innovation networks across countries and across different network functions. The study argues that investigating multi-stakeholder innovation network characteristics targeted by the MSP using a network approach in early implementation can contribute to better performance in generating and scaling innovations, and that funding can be an effective implementation tool in developing country contexts.

## Introduction

Stakeholder involvement is essential to overcome complex agricultural and environmental problems and achieve development outcomes. Multi-stakeholder platforms (MSPs) are seen as an effective vehicle to support stakeholder involvement in multi-stakeholder processes [1–4]. For instance, in agricultural innovation systems, MSPs are expected to contribute to creating an enabling environment for technological and institutional innovation, and to facilitate effective up- and out-scaling of these innovations to achieve development impact [3]. The increasing popularity of multi-stakeholder and innovation platforms in agriculture and development fields shows optimism about the possibilities for MSPs to foster change and development deliberately and effectively [3, 5]. However, bringing together diverse groups of stakeholders in a platform will not automatically lead to innovation or scaling; MSPs have also been reported to fail in delivering their objectives [6–8].

MSPs bring together a group of stakeholders working in different sectors. Depending on the issue at stake, these stakeholders can include farmer, private sector, government, research, and extension actors [9]. In the course of the MSPs, participating stakeholders, i.e. individuals, groups, and organizations [8] (hereafter MSP stakeholders), come together and “get things done” [10]. What is “done” depends on stakeholders’ characteristics such as their capacity and motivation [11] and how they integrate into multi-stakeholder innovation networks (hereafter innovation networks) that give them access to different benefits such as information, markets, and finance [12]. Integration into these innovation networks is effected through other stakeholders in these networks, i.e. innovation network stakeholders, and depends on the connections among them [12] both in and outside MSPs. In other words, the characteristics of innovation network stakeholders affect what is done in MSPs and therefore also the MSPs’ contributions to innovation and scaling.

The objective of this paper is to investigate the effects of MSPs on innovation networks. We focus on three characteristics–size, connectivity, and configuration–of innovation networks to study the changes and explore the factors contributing to these changes. We use three cases, one each from Burundi, Democratic Republic of Congo (henceforth referred to as DRC), and Rwanda, implemented by a CGIAR research programme called Integrated Systems for the Humid Tropics (Humidtropics) for more than a year. The paper addresses two research questions: What changes do MSPs trigger in the characteristics of innovation networks? What other external factors shape the changes triggered by MSPs in innovation networks? The implications for the contributions of MSPs to innovation and scaling without empirical testing are then discussed.

## Concepts, methods and analysis tools

### Empirical framework

#### Description of MSPs and Humidtropics programme

**Fig 1:**
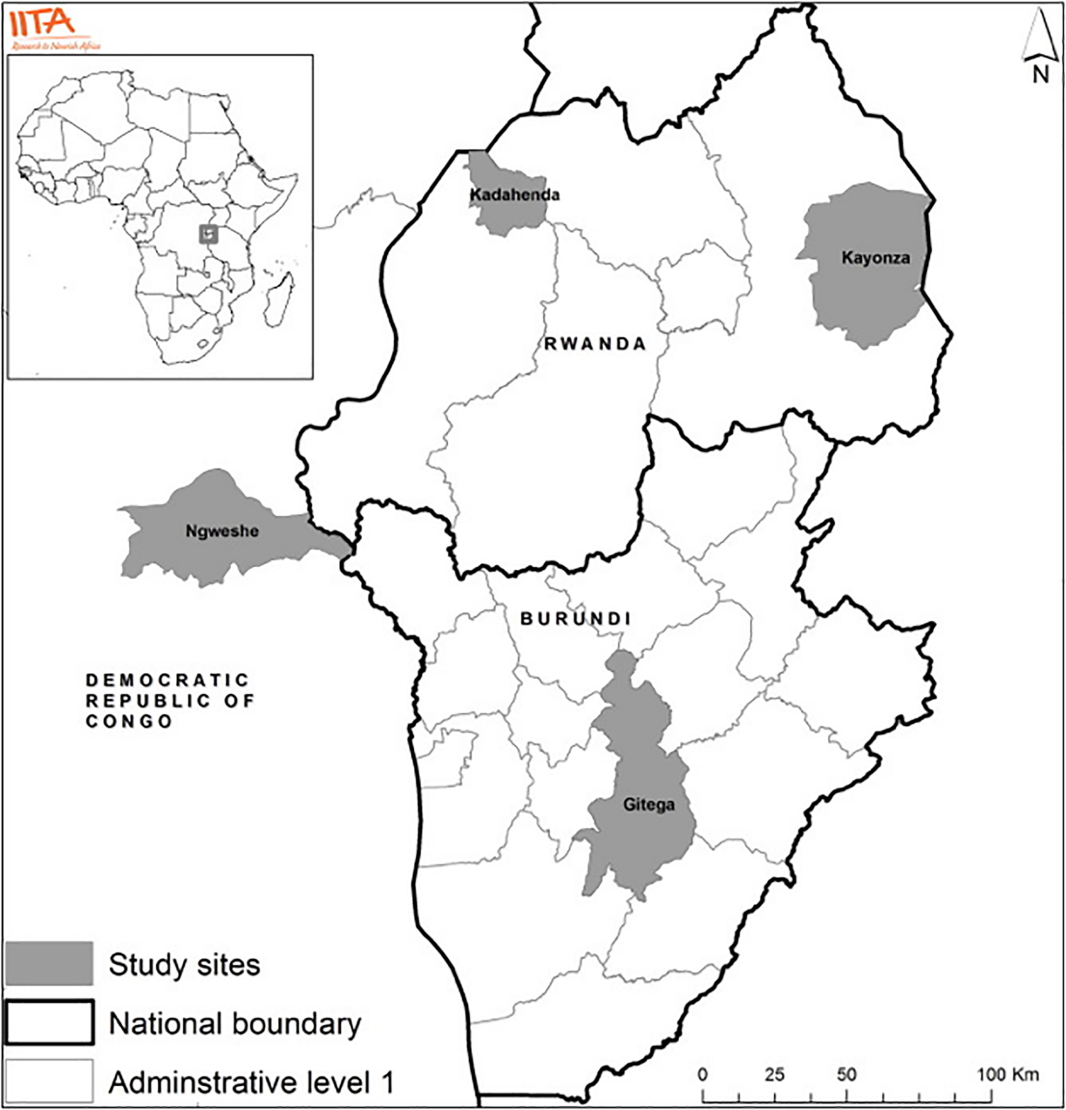
Operational areas of the multi-stakeholder platforms. Source: [13].

The MSPs studied in this paper started to be operationalized in Burundi, DRC, and Rwanda in mid-2013. They were initiated in May 2013 in Bukavu, DRC, and in July 2013 in Bujumbura, Burundi, and in Kigali, Rwanda. MSP field-based activities were implemented in Gitega province of Burundi, Ngweshe in DRC, and Kadahenda and Kayonza in Rwanda (Fig 1). The MSPs targeted multiple goals: improving income and nutritional status of the poor, improving farm productivity without causing environmental degradation, empowering women and youth, and improving the innovation capacity of agricultural innovation systems. They aimed to optimize the achievement of these goals by investigating and dealing with synergies and trade-offs among the goals.

In each country, MSP activities were organized through multiple events in which different numbers and types of stakeholders participated. These events included research events such as setting up, monitoring field trials, and researcher meetings; management events like platform event preparation, sub-groups, and reflection meetings; and other events such as capacity building activities, promotion, and fundraising events for the platform. These events were organized mostly by the lead organization of the Humidtropics programme or in some cases by other MSP participants. The number and sequence of the events varied in each country.

In addition to organizing and funding events, the Humidtropics programme (1) identified MSP facilitators and funded the salaries of these facilitators, (2) provided inputs to support some of the activities identified in the MSP, (3) funded small research projects prioritized by the MSPs, i.e. platform lead research projects, (4) supported or established groups or innovation platforms to better organize activities located in places distant from the capitals (where MSPs events mostly take place), and (5) managed the administration, monitoring, and evaluation of the small research projects and managed other expenses incurred for the MSPs.

Stakeholders were initially selected through a combination of two approaches. The first approach was to send invitations to the representatives of the organizations with which the intervention managers had a long history of collaboration. These included central and local government actors, international organizations, and NGOs specialized in the sector intervention. The second approach was to organize open events and calls to encourage the involvement of stakeholders operating in the target locations. Stakeholders enrolled by these two methods were given the same support in their involvement in the intervention events to minimize the bias of positive selection of stakeholders with a history of collaboration.

#### Data collection and cleaning

Data were gathered through written surveys in Burundi, DRC, and Rwanda in August 2014 (t = 1) and in October 2015 (t = 2). For both surveys (t = 1 and t = 2), the MSP participants were asked to provide the following information: (1) name, gender, age; (2) all organizations/institutes/companies with which they were affiliated; (3) all organizations in their professional network with which they collaborated; (4) the five organizations from their network that they found to be the most important for knowledge exchange; and (5) the five organizations from their network that they found to be the most influential (S1 and S3 Files). During the second survey, seven questions relating to the functioning of the MSP were added. These included three questions on whether the MSPs had enforced their collaboration, knowledge exchange, and influence spread (ranking agreement on a 5-point scale); two questions on which types and scales of organizations they think more effective in improving capacity to innovate and upscale innovations, i.e. key organizations; and two questions on connections of key organizations among themselves and other organizations influential (S2 and S4 Files). The data collected by the initial round of surveys was published in another research paper by Hermans et all [14].

Data were entered and cleaned by researchers and the MSP facilitators to enable the matching of organizational abbreviations and full names, to synchronize French and English abbreviations of organization names, and to decipher handwriting and misspelling of names and abbreviations. Where necessary, the organization names were validated through online search.

The accounts of the implementing organization were used to identify the funding allocated to individual organizations and different events. Events organized by the MSPs and the activities targeted by them were identified by using an event-based monitoring and reporting system: learning system for agricultural research for development (LESARD) [15]. The co-authors of this paper also attended MSP events. Our participatory observations in these MSP events contributed to our understanding of the data and results.

#### Data analysis

This paper provides two snapshots of different innovation networks in two different time periods. We used a two-tiered approach in the analysis. Firstly, a social network approach was used to investigate the changes in the size, connectivity, and configuration characteristics of the innovation networks in Burundi, DRC, and Rwanda. Network analysis was used to calculate network statistics for collaboration, knowledge exchange, and influence spread networks using the concepts and measurements presented in Table 1. Size and tie information provided by the network statistics was complemented with network maps to further explore the changes in configurations of collaboration, knowledge exchange, and influence spread networks. Network properties were analysed and visualized using Gephi v.0.9.1 [16].

**Table 1:** Concepts and measurements in network analysis.

Concept	Mathematical notation	Definition
Graph	*G (N*, *£)*	Model for a network with a set of nodes connected by a set of ties
Node	*N = [n*_*1*_,*n*_*2*_,*n*_*3*_, *…*,*n*_*g*_*]*	Organizations depicted in the graph
Tie	*£ = [l*_*1*_*l*_*2*_,*l*_*3*_, *…*, *l*_*L*_*]*	Undirected connection between nodes
Size	*G*	The number of nodes in the graph
Degree of a node	*Size of £*	The number of ties in a node

Secondly, we used logistic regressions to explore statistically the factors that contributed to the changes in the characteristics of the networks. Variables entering the models were selected by forward stepwise selection using a likelihood-ratio test [17]. We explained (1) the dichotomous continuation status of the ties in the collaboration, knowledge exchange, and influence spread networks at the initial survey, i.e. continue or drop, and (2) the factors that differentiate the characteristics of the ties joining the networks from the ones that were there at both times, using the factors presented in Table 2. We used SPSS v.23 for the logistical models.

**Table 2:** Factors and variables used to explore the changes in multi-stakeholder network characteristics.

Factors	Variables	Variable descriptions	Variable values
**Institutional environment**	Country of operation	The country where the organizations operate, taking a different integer value for Burundi, DRC, and Rwanda	1: Burundi 2: DRC 3: Rwanda
**Initial innovation network characteristics**	Number of organizations	Number of organizations in the innovation networks	Positive integers
	Number of connections	Number of connections between the same organizations in the existing innovation networks	Positive integers
	Type configuration	A variable taking a different value for each tie	1: Business 2: Farmer 3: NGO/CSO 4: Government 5: Research/ Extension/ Education
	Scale configuration	A variable taking a different value for each tie	1: District 2: Province 3: National 4: Supranational
**Types of problems targeted by MSP**	Change in the number	Change in the number of targeted problems in the MSPs, including improving farm productivity, income, nutritional status, environmental degradation, empowering women and youth, and capacity of innovation systems	Integers where each problem theme has the same weight
**Funding provided by MSP**	To organizations	Amount in US Dollars provided to some selected organizations	Continuous in Dollars
	To events	Share of the events that MSP manager organization fully funded during the MSP (scale)	Percentages
	To collective decisions	A variable for showing provision of platform lead funding (PLF)	0: No PLF 1: Yes PLF
**Type of activities (events) in the MSP**	Number of events	Number of events recorded by the MSP	Positive integers
	Share of innovation-generation events	Share of the innovation-generation events in the MSP	Percentage
	Share of innovation-diffusion events	Share of the innovation-diffusion events in the MSP	Percentage
	Share of innovation-use events	Share of the innovation-use events in the MSP	Percentage
	Share of management events	Share of the management events in the MSP	Percentage
	Share of process backstopping events	Share of the backstopping events in the MSP	Percentage

### Conceptual framework

#### Typology of stakeholders in livelihood and innovation systems based on their involvement in interventions with MSPs

**Fig 2:**
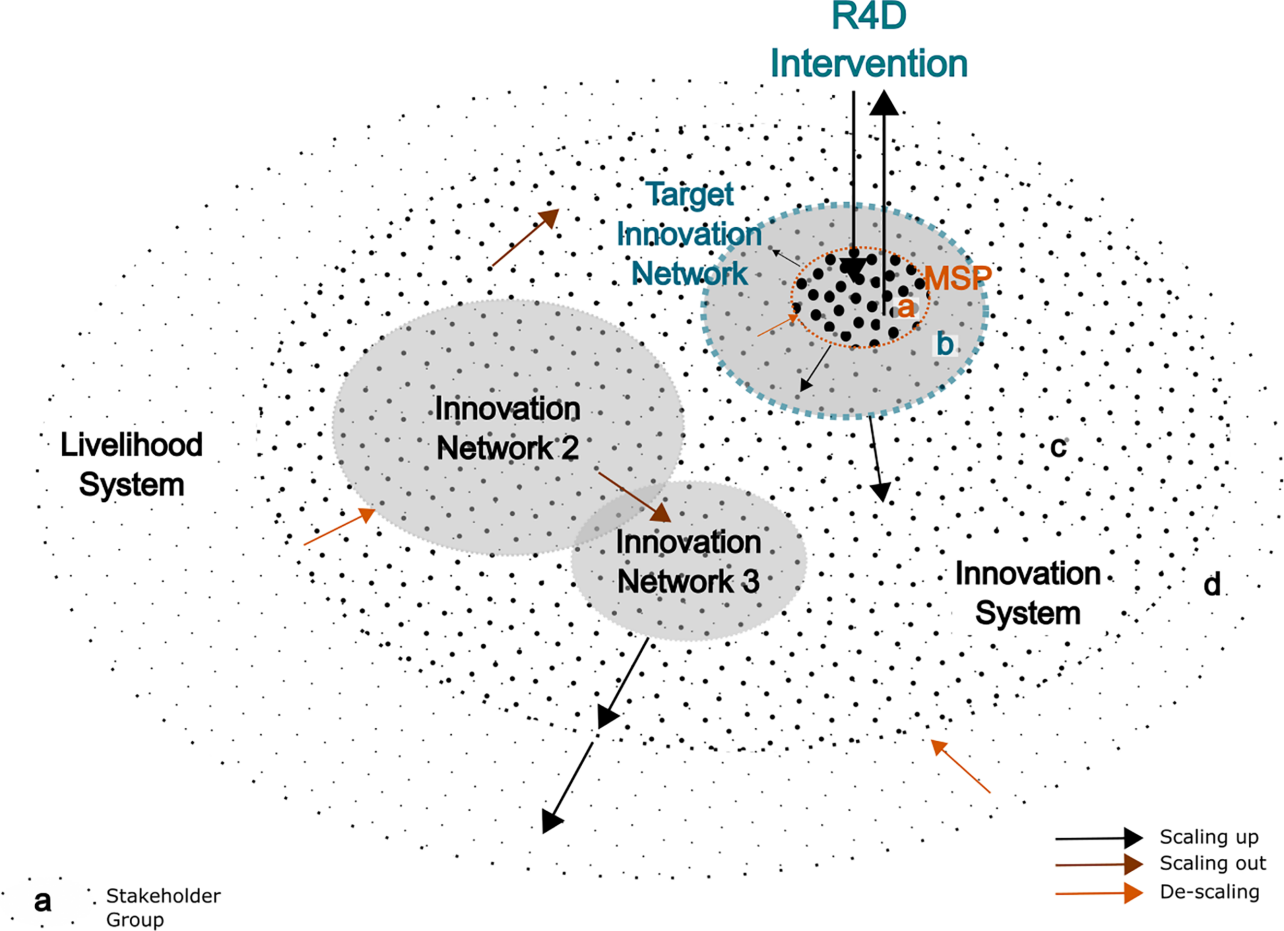
Stakeholders in livelihood and agricultural innovation systems. Dots represent different stakeholders and the circles surrounding them represent the group of stakeholders operating in multi-stakeholder platform (a), innovation network (b), innovation system (c), and livelihood system (d). MSP targets a sub-group of an innovation network (orange circle) with its events and influences, and is influenced by, the characteristics of that network (blue circle).

Stakeholders in livelihood systems differ in their involvement with MSPs and with the interventions that MSPs organize. A subset of stakeholders participate in the intervention platform and have a direct chance of influencing the MSP’s agenda and events (Table 3). A second group of stakeholders are involved in the intervention like the MSP stakeholders but are not involved in the platform. Therefore, they can influence the agenda and events of the intervention but not as directly as the MSP stakeholders. As the second group of stakeholders collaborate with the MSP stakeholders in developing the innovations targeted by the intervention, we refer to the combination of MSP stakeholders and the second group as innovation network stakeholders (Table 3). A third group of stakeholders are not involved in the intervention but can influence the impact of the innovation on livelihood systems. They can be collaborating with the stakeholders in the innovation network, or they may be part of a distinct innovation network whose members are connected to the intervention’s innovation network (Fig 2). As the stakeholders in the innovation network and the third group of stakeholders define the boundaries of the stakeholders who can influence the impact of the innovation on livelihood systems, we define their combination as a new stakeholder group, innovation system stakeholders. Finally, there is a fourth stakeholder group, who are not involved and do not have any influence on the agenda and events of the intervention. Moreover, they do not have any direct influence on the impact of the intervention on livelihood systems. They constitute all the stakeholders in the livelihood system other than the stakeholders in the innovation system.

**Table 3:** Typology of stakeholders in livelihood and innovation systems based on their involvement in interventions with MSPs and the influence of the intervention on livelihood systems.

Stakeholder group as a whole	Involvement in the intervention with MSP	Involvement in the MSP	Influence on the agenda and events of the intervention with MSP	Influence on the impact of the intervention on livelihood systems
**MSP (a)**	Yes	Yes	Direct	Direct
**Innovation network (b)**	Yes	No	Indirect	Direct
**Innovation system (c)**	No	No	None	Direct
**Livelihood system (d)**	No	No	None	Indirect

In terms of stakeholder types based on value-chain functions, MSP stakeholders consisted mostly of researchers in the cases studied. They also included government representatives, technical staff working on targeted innovations, and NGO staff such as farmer representatives working in the locations targeted by the interventions. The innovation networks surrounding the MSP stakeholders included central government actors, UN organizations, and the managers of the organizations’ MSP stakeholders, located in bigger cities or in some cases abroad. Provincial and national policymakers and innovation networks organized around other projects were members of the innovation systems in the cases investigated. In almost all the MSPs investigated, there were a few other interventions working on innovations related to the cases on which we focused. Typical examples were interventions focusing on nutrition aspects or marketing aspects of the focus crops in the MSPs studied. Some of the MSP or innovation network stakeholders involved in the cases we investigated were also members of the innovation network of the other interventions (Fig 2).

#### Network-based stakeholder typology, scaling out, and scaling up

The innovation system literature commonly describes the dissemination of the use of innovations among different stakeholder groups as *scaling out*, whereby innovations developed by livelihood interventions are used in another geographical location [18, 19], or *scaling up* whereby innovations are institutionalized and are commonly used at different geographical locations and in different institutional setups [20–23].

Both definitions are based on geographical location, and scaling up also includes an element of institutional embedding. Spreading the use of innovations from MSPs to outside (Fig 2) implies a change in functional stakeholder types, such as from researchers to policymakers, and mostly entails institutional embedding. Therefore, such movements can be considered as scaling up. Spreading an innovation between the same stakeholder type, such as from one innovation network to another, can be considered as scaling out as it does not imply institutional embedding. The network-based typology captures both scaling up and scaling out dimensions of innovation processes (Fig 2). In addition, it captures the cases of *descaling*, where innovations become less used by similar types of actors or the institutional support behind the innovations is lost.

#### Multi-stakeholder platforms as network interventions

Social networks influence individuals’ practices in various aspects of life, including personal and work practices, and they can be leveraged to achieve behavioural and social change. Network interventions are interventions that use the leverage of these social networks purposefully [24] and are shown to improve the dissemination and spreading of innovations [25]. Understanding the impact of interventions such as MSPs requires interaction between the actors and their dynamics, i.e. their networks [26]; and MSPs’ aim to enhance an enabling environment for the creation, up-scaling, and out-scaling of innovations [3] requires behavioural and social changes. Therefore, MSPs can be considered as network interventions. Moreover, studying network interventions can contribute to better understanding the complexity and multi-dimensionality of innovation processes [27] and effectiveness factors, and to making better informed decisions about stakeholder strategies [28]. It also offers governments new opportunities to stimulate agricultural innovation [29]. Thus, we chose a network intervention approach to study changes triggered by MSPs in innovation networks.

#### MSP factors affecting characteristics of multi-stakeholder innovation networks across time

The MSP literature reports several performance factors. Firstly, the role of the institutional environment in which innovation networks and MSPs operate has often been found [3, 4] to be a factor that influences how MSP perform. Moreover, funding has been identified as an important performance factor for MSPs [6, 8, 30]. A further factor for the performance of multi-stakeholder interventions such as MSPs is the type of problem targeted by them [31, 32]. In addition, some types of activities (e.g. entrepreneurial) have been reported to play a role in innovation processes [33] and influence the performance of MSPs [26].

Some other performance factors reported in the literature depend on the initial conditions in the innovation networks. One such factor is the initial strength of the connections [34]. Another is the type of stakeholder in innovation networks. Participation by farmers, NGOs, research organizations, government actors, and the private sector has been reported to make different contributions to MSP performance [1, 8, 35]. In addition, the scale at which a stakeholder operates affects the scaling potential of an innovation network in that innovation system [3, 36]. Therefore, we consider the number of existing organizations and connections, and the change in type and scale of configurations of the innovation networks. In brief, in this paper, we focus on the institutional environment (1) of the country in which the innovation system, the innovation networks, and the MSP operate (2), the number of organizations and strength, type, and scale of existing connections in these innovation networks (3), type of activities in which MSPs engage (4), changes in MSP funding (5), and problems on which MSPs focus (6).

#### Multi-stakeholder network characteristics influencing innovations and scaling in agricultural innovation systems

A first characteristic of innovation networks that influences innovations and scaling is the size of the network. A bigger innovation network will imply a stronger position vis-à-vis other innovation networks [37], and innovations are considered to have a better outreach if the size of the networks in which they operate is larger [23]. A second characteristic reported to be influential in innovations and scaling is the connectivity of the stakeholders in innovation networks. As the connectivity of innovation networks has been shown to be positively related to the outreach of the innovations and the speed of innovation diffusion [23, 24], MSPs can be more effective if they trigger an increase in the connectivity of innovation networks. In other words, the size and the connectivity of an innovation network influence the likelihood of successful innovation and scaling.

The characteristics of (1) overall collaboration [17, 30], the general category of working together without specification, and two major aspect of collaboration (2) knowledge exchange [29] and (3) influence spread [11, 17] between stakeholders of innovation networks are considered to play a role in innovation and scaling. In brief, changes in collaboration, knowledge exchange, and influence spread in the innovation networks over the course of MSPs can elucidate the effects of MSPs on innovation and scaling. Therefore, in this paper, we focus on the size and the connectivity of innovation networks in terms of collaboration, knowledge exchange, and influence spread (hereafter innovation network functions). We support the results with network maps to further explore change in the network configurations.

## Results

### Characteristics of the Humidtropics multi-stakeholder platforms

The MSPs in Humidtropics were organized in Burundi, DRC, and Rwanda using the same management approach. The Humidtropics programme identified and funded facilitators, provided backstopping for events and innovation platforms, managed MSP administration, and provided funding in all the country cases. However, there were several differences in the MSPs across the countries, such as individual funding provided to individual organizations. Other differences are presented in Table 4.

**Table 4:** Differences in MSPs in Burundi, DRC, and Rwanda. Percentages represent the characteristics of the factors between surveys. DRC received the least funding support, and Rwanda received the most. Types of problems targeted by the MSPs increased in Burundi and DRC and stayed the same in Rwanda. Rwanda has the highest number and highest ratio of innovation-generation, innovation-diffusion, and innovation-use events.

	Burundi		DRC		Rwanda	
Funding	t1	t2	t1	t2	t1	t2
***Platform lead project***	Yes	No	Yes	No	Yes	Yes
***Share of events exclusively funded***	90%		66%		89%	
**Targeted problems/ Goals**						
***Agricultural productivity***	Yes	Yes	Yes	Yes	Yes	Yes
***Income***	No	Yes	No	Yes	No	No
***Nutrition***	No	Yes	No	Yes	No	No
***Gender***	No	Yes	Yes	Yes	No	No
***Innovation capacity***	No	No	No	No	No	No
**Activities in the MSP**						
***Number of events***	34		54		99	
***Share of innovation-generation events***	12%		9%		38%	
***Share of innovation-diffusion events***	0		0		6%	
***Share of innovation-use events***	3%		2%		6%	
***Share of management events***	32%		46%		26%	
***Share of process backstopping***	44%		20%		19%	

### Changes in multi-stakeholder innovation network characteristics

**Fig 3:**
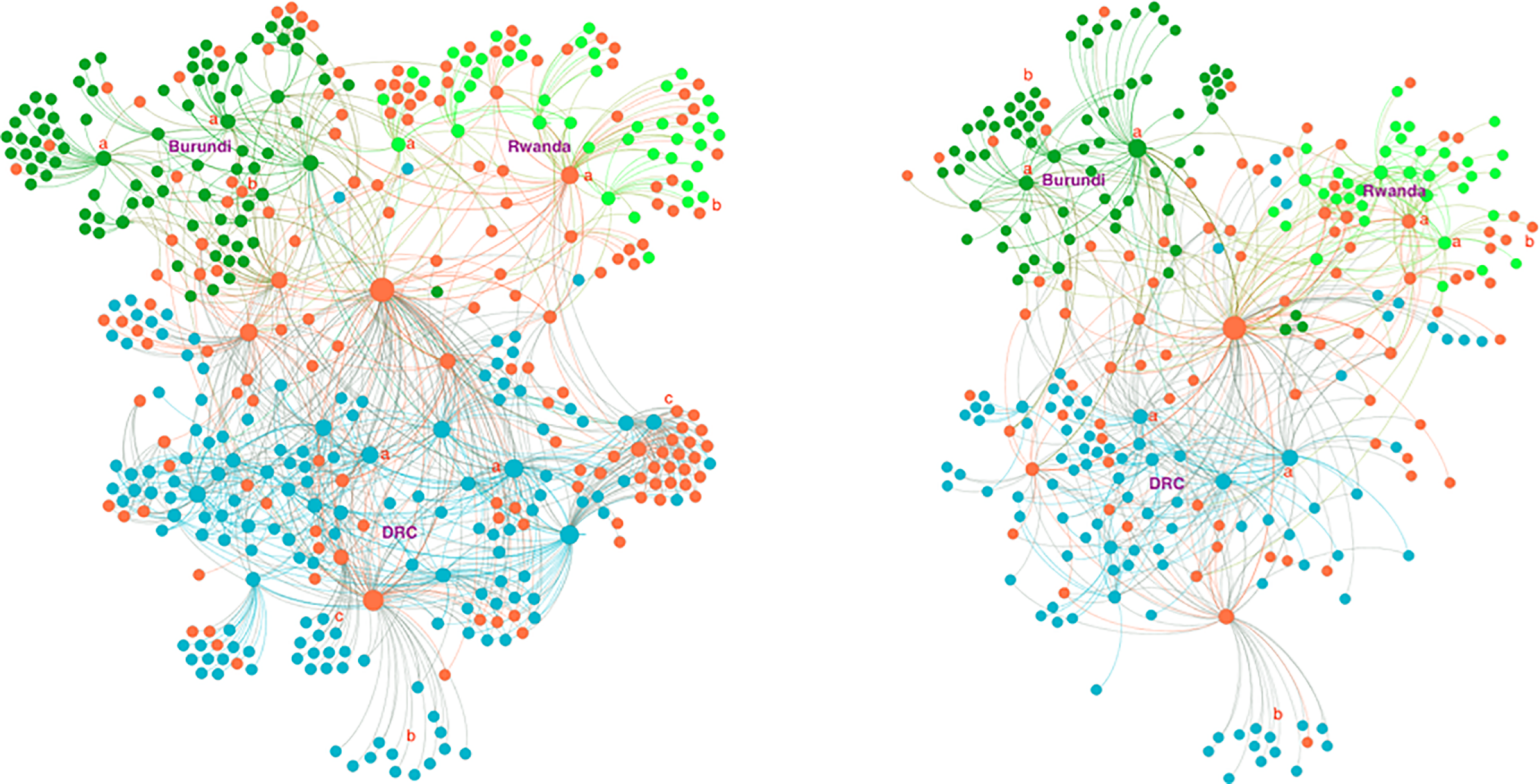
**Maps of multi-stakeholder innovation networks in Burundi, DRC, and Rwanda in t1 (left) and t2 (right).** Node size represents the degree centrality. Dark green (upper left) nodes represent organizations based in Burundi, blue (below) represents DRC, light green (upper right) Rwanda, and orange supranational organizations. Dark green coloured ties represent organizational connections in Burundi, blue represents DRC, and light green represents Rwanda. Collaboration in innovation networks was positioned around locally central actors (a) in each country and contained sub-clusters with both national and supranational organizations (b). After the MSP, some sub-clusters (c) left the collaboration.

Changes in the characteristics of collaboration, knowledge exchange, and influence spread presented both similarities and differences. In terms of network size and connections, Burundi and Rwanda experienced similar changes, and DRC experienced different ones (Table 5). Most of the MSPs (Fig 3A) maintained their intermediator role between the organization managing the MSPs and the other stakeholders, which are combinations of national and international organizations (Fig 3B). However, some MSPs left the collaboration (c). In each country, the number of sub-clusters around a single organization decreased substantially. The sub-clusters decreased either because some MSPs dropped out (c) or because of network closure in the innovation network, especially in Rwanda (Fig 3).

**Table 5:** Changes in the collaboration, knowledge exchange, and influence spread characteristics of multi-stakeholder networks in Burundi, DRC, and Rwanda.

Network characteristics		Burundi			DRC			Rwanda		
		T1	T2	Δ	T1	T2	Δ	T1	T2	Δ
**Collaboration**	**Size**	120	100	-17%	246	147	-40%	103	76	-26%
	**Ties**	202	183	-9%	844	314	-63%	153	188	23%
	***With 1***	*183*	*129*	*-30%*	*701*	*256*	*-63%*	*27*	*139*	*9%*
	***With 2+***	*19*	*54*	*184%*	*143*	*58*	*-59%*	*26*	*49*	*88%*
**Knowledge exchange**	**Size**	31	36	16%	34	24	-29%	23	25	9%
	**Ties**	71	77	8%	189	69	-63%	43	79	84%
	***With 1***	*58*	*60*	*3%*	*152*	*60*	*-61%*	*37*	*55*	*49%*
	***With 2+***	*13*	*17*	*31%*	*37*	*9*	*-76%*	*6*	*24*	*300%*
**Influence spread**	**Size**	27	39	44%	41	15	-63%	22	21	-5%
	**Ties**	50	83	66%	207	51	-75%	43	67	56%
	***With 1***	*50*	*64*	*28%*	*170*	*47*	*-72%*	*37*	*56*	*51%*
	***With 2+***	*0*	*19*	*N*.*A*.	*37*	*4*	*-89%*	*6*	*11*	*83%*

#### Collaboration networks

Across all the countries, the size of collaboration networks decreased between the observation periods at t1 and t2 (Table 5). The highest decrease was observed in DRC with 40%, followed by Rwanda 26% and Burundi 17%. Apart from Rwanda, the number of collaboration connections, or ties, also decreased. Across the countries, multiple ties between the same organizations decreased less than the single ties in the collaboration networks. In Burundi and Rwanda, the number of such multiple ties increased by 184% and 88%, respectively.

#### Knowledge exchange networks

**Fig 4:**
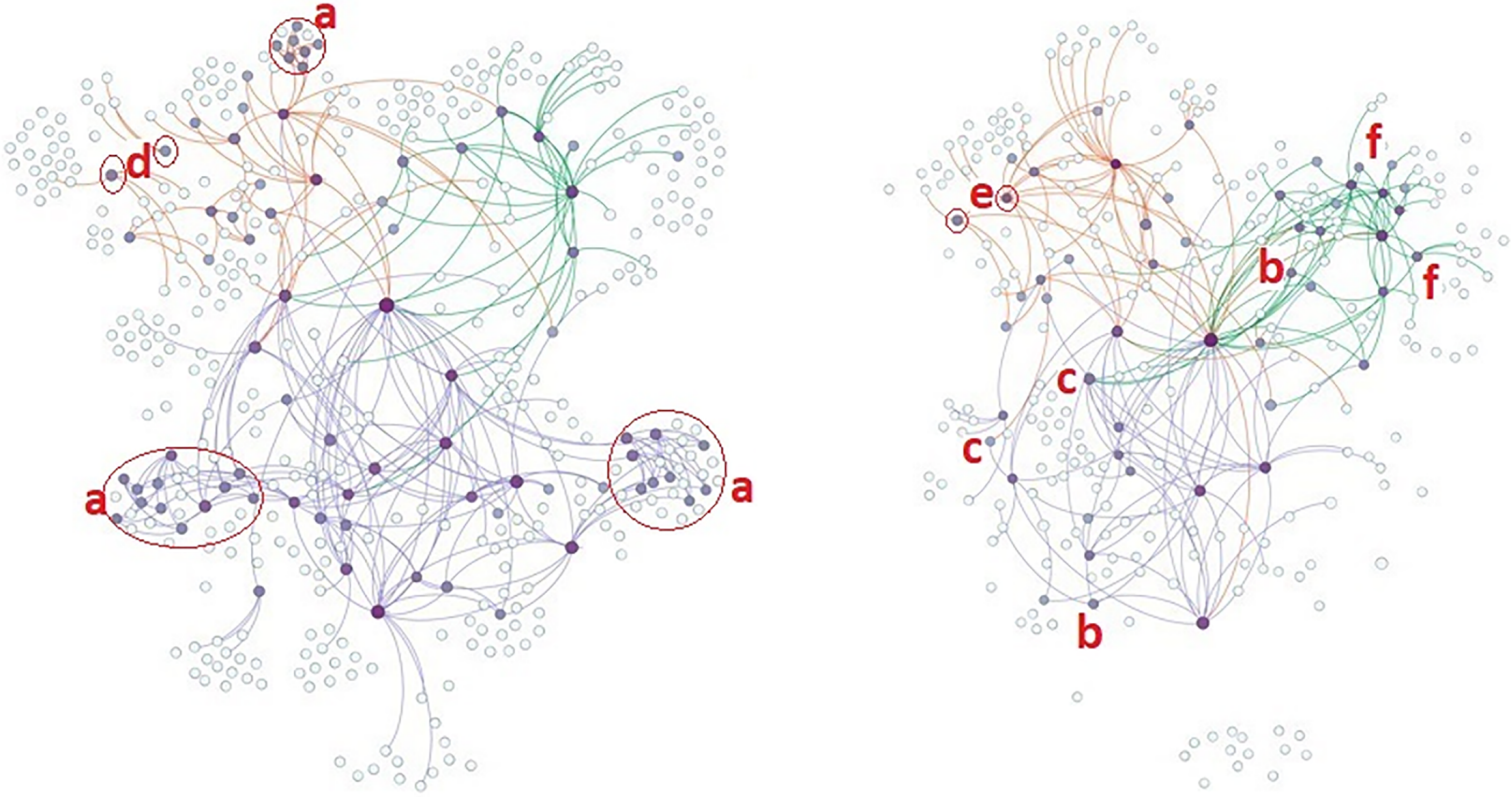
**Knowledge exchange in innovation networks in Burundi, DRC, and Rwanda in t1 (left) and t2 (right).** Node size and boldness represent the degree of knowledge exchange centrality. White nodes are parts of innovation networks but not knowledge exchange. An orange tie colour represents connections in Burundi, purple in DRC, and green in Rwanda. During the MSP, all knowledge exchange clusters that were not initially connected to the lead organization (a) left the network. New knowledge exchange connections were generated either by participation of national organizations (b) or by establishing cross-boundary connections (c). Isolated clusters in the initial network (d, e) connected to the main clusters, and some new organizations (f) joined the network.

Knowledge exchange in Burundi, DRC, and Rwanda experienced different changes in comparison to changes in collaboration. In Burundi and Rwanda, the number of organizations exchanging knowledge increased despite the contraction in collaboration (Table 5). The number of organizations exchanging knowledge increased from 31 to 36 in Burundi (nodes with orange ties–Fig 4) and from 23 to 25 in Rwanda (nodes with green ties–Fig 4). In DRC, the number of organizations exchanging knowledge decreased from 34 to 24. Similarly, knowledge exchange ties and the ratio of multiple ties increased in Burundi and Rwanda but decreased in DRC. However, in all three countries, the ratio of the organizations exchanging knowledge in innovation networks increased, as the contraction of the knowledge exchange was smaller than the collaboration. The ratio of the organizations exchanging knowledge increased from 26% to 36% in Burundi, 14% to 16% in DRC, and 23% to 33% in Rwanda.

Across the countries, the MSPs’ managing organization increased its knowledge exchange connections. All knowledge exchange clusters not directly linked to the managing organization (Fig 4A) dropped out in Burundi and DRC. The expansion of the knowledge exchange was attributable to the participation of new national organizations (Fig 4B) as well as to the establishment of cross-boundary connections with organizations operating in the other two countries in the region (Fig 4C). Other changes in the knowledge exchange happened either through existing isolated organizations (Fig 4D) joining the knowledge exchange (Fig 4E) or some new organizations joining the innovation network and the knowledge exchange (Fig 4F).

#### Influence spread networks

**Fig 5:**
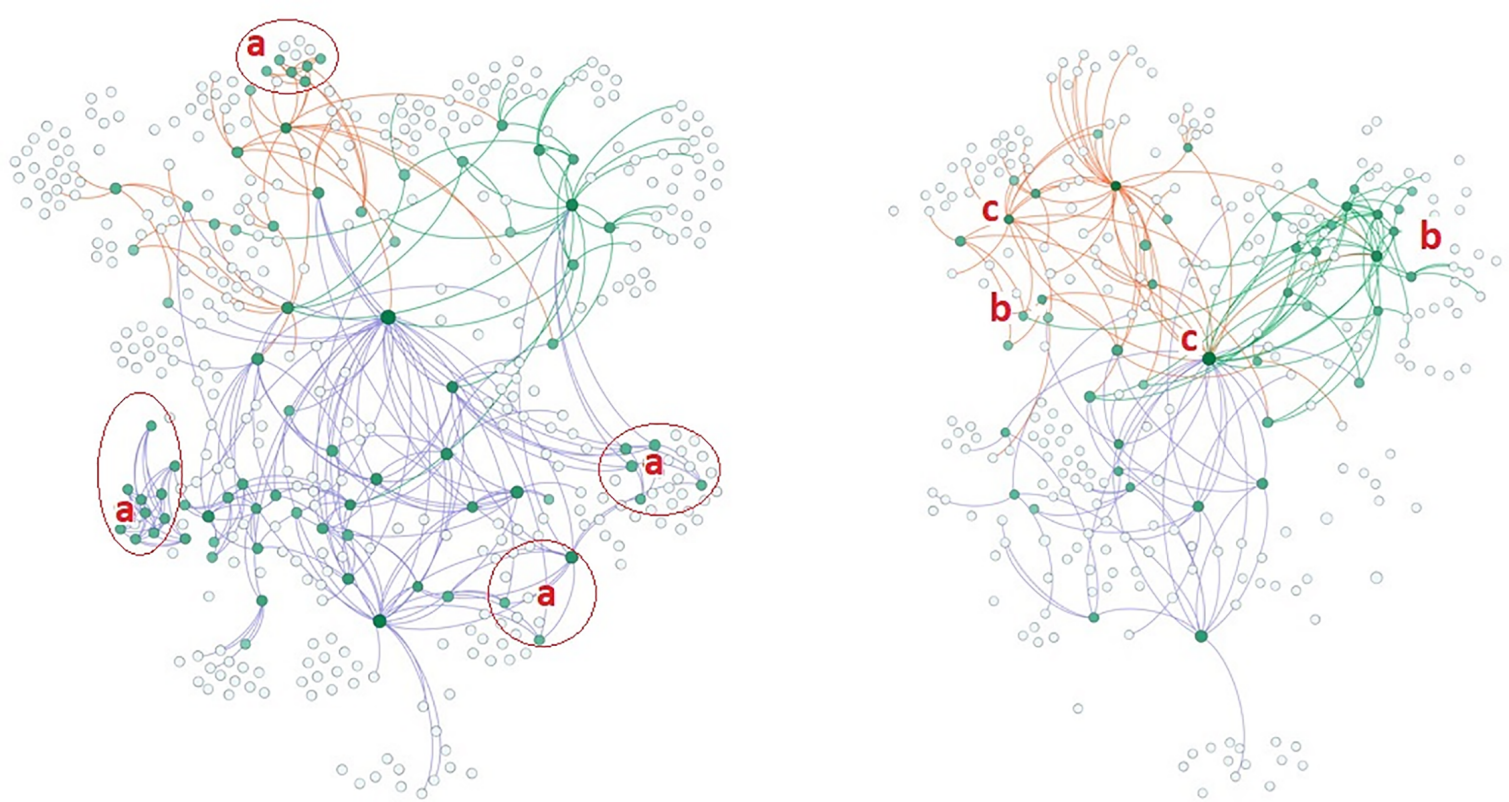
**Influence spread in innovation networks in Burundi, DRC, and Rwanda in t1 (left) and t2 (right).** Node size and boldness represents the degree of influence centrality. White nodes are parts of innovation networks but not influential. An orange tie colour represents connections in Burundi, dark blue in DRC, and green in Rwanda. During the MSP, some existing influence clusters (a) left the networks, some organization (b) joined the influence spread networks, and some existing organizations (c) increased their influence.

Influence spread networks experienced different changes in the countries. Whereas the number of influential organizations increased in Burundi by 44%, it decreased by 5% in Rwanda and by 63% in DRC (Table 5). Most of the contraction in Burundi and DRC was attributable to the disappearance of some influence clusters (Fig 5A). An increase in the MSP managing organization’s influence ties (Fig 5C) was the major driver of the increases in mean degree of influence in Burundi and Rwanda. In Burundi and Rwanda, the participation of small groups (Fig 5B) of influential organizations in the innovation networks and, in Burundi, the increase in the influence ties of some organizations (Fig 5C) supported the major driver. However, no such continuing influential organization was observed in DRC.

### Factors influencing multi-stakeholder innovation network characteristics

Factors explaining the changes in the configurations of the collaboration, knowledge exchange, and influence spread networks differed in terms of the two major changes observed: i) incumbent stakeholders leaving and ii) new stakeholders joining the networks (Table 6). For both, correctly predicted percentages were more than 80%.

**Table 6:** Results of logistic regressions explaining the factors that affect multi-stakeholder innovation network configurations. Initial characteristics of the innovation networks and funding were significant both in term of incumbent stakeholders’ decision to stay and new stakeholders’ decision to join.

Factors and Variables		Incumbents staying (Leave: 0, Continue: 1)						New stakeholders joining (Incumbent: 0, New: 1)					
		Collaboration		Knowledge exchange		Influence spread		Collaboration		Knowledge exchange		Influence spread	
		Exp (β)	Wald	Exp (β)	Wald	Exp (β)	Wald	Exp (β)	Wald	Exp (β)	Wald	Exp (β)	Wald
**Innovation network characteristics**	***Number of organizations at t1***			.85**	23.89	.93**	74.9						
	***Number of connections at t1***	*3*.83**	*54*.*01*	*2*.64**	*9*.*55*			.47**	*41*.*30*	.17**	*22*.*89*	.28**	*19*.*93*
	***Type configuration***												
	***Business***	*2*.35*	*4*.*90*										
	***Government***	*2*.08**	*6*.*89*					.50**	*9*.*70*				
	***NGO***	*1*.86*	*5*.*90*							.42*	*4*.*09*		
	***Research***	*2*.67**	*8*.*34*							.19**	*9*.*40*		
	***Scale configuration***												
	***District***	.18**	*11*.*11*										
	***Provincial***	.54*	*4*.*73*			.30*	*4*.*3*						
**Funding provided by MSP**	***To organizations***	*1*.04**	*13*.*34*	*1*.04**	*6*.*30*	*1*.05**	*7*.*65*						
	***To events***	.01**	*71*.*69*					*38*.51**	*43*.*56*	*196*.59**	*51*.*97*	*47*.79**	*53*.*03*
**Types of problems targeted by MSP**	***Change in the number***	*0*.63**	*11*.*05*					*1*.38*	*6*.*00*				
**Model statistics**	***Log likelihood***	*680*.*91*		*194*.*22*		*169*.*50*		*554*.*55*		*172*.*88*		*160*.*56*	
	***Cox & Snell*** ***R Square***	.*56*		.*53*		.*56*		.*44*		.*46*		.*44*	
	***Nagelkerke*** ***R Square***	.*75*		.*70*		.*75*		.*58*		.*62*		.*59*	
**Hosmer and Lemeshow Test**	***Chi-Square***	*4*.*73*		*3*.*23*		*0*.*65*		*7*.*24*		*5*.*50*		*4*.*93*	
	***df***	*8*		*5*		*3*		*8*		*8*		*3*	
	***Significance***	.*79*		.*66*		.*89*		.*51*		.*70*		.*18*	
**Predicted**	***Correct*** ***percentage***	*89*.*2*		*88*.*8*		*89*.*7*		*81*.*9*		*85*.*3*		*84*.*6*	

All models are significant with p values less than 0.01.

(*) and (**) denote significance level for individual factors at 0.05 and 0.01.

Country of operation and number of problems targeted at t1 were not significant in any of the innovation networks. Farmers belonging to type configuration and national and supranational organizations in scale composition were not significant for any innovation networks.

None of the event variables, i.e. number of events; share of innovation-generation, -diffusion, and -use events; aggregation of all innovation events; management or backstopping events, was significant. As Platform Lead Small Research was provided only to Rwanda at t2, the variable was highly correlated with country, and it was dropped from the models.

## Analysis and discussion

### Common changes in multi-stakeholder innovation networks

Our study indicated two major common aspects of change in innovation networks following MSPs: heterogeneity of change in innovation network functions and centralization of innovation networks. Our results showed that the changes in size and connectivity depended on the specific innovation functions. Whereas network size and the number of ties decreased in collaboration networks, they increased in knowledge exchange and influence spread networks (Table 5). Moreover, the changes in collaboration varied more not only across countries, but also in terms of factors that play a significant role in the changes. Changes had higher variability across countries, and the number of significant factors was higher in collaboration networks than in knowledge exchange and influence spread networks (Table 6). This confirms the distinction–suggested by the literature on agricultural innovation systems [17, 29] as well as other sectorial innovation systems [11, 38]–between the changes in different functions fulfilled by innovation networks.

Secondly, our data show that MSPs did not necessarily lead to decentralized networks where different innovation network stakeholders have high collaboration, knowledge exchange, and influence connections. On the contrary, the MSPs’ lead organization (represented by the largest node in Fig 3) increased its knowledge exchange (Fig 4C) and influence centrality (Fig 5), whereas the majority of the other influential and central knowledge exchange organizations disappeared from the innovation networks (Figs 4 and 5). Although a central position for the MSPs’ lead organizations is neither rare nor necessarily problematic [32], it indicates that their point of view will be more represented in the networks [39], and the needs and participation of some stakeholders will be undermined [26]. This is a risk for innovation and scaling, as the influence of MSPs’ lead organizations can disrupt the existing networks, can outcompete other organizations from the networks [40], and create a situation where stakeholders are willing to collaborate with the lead but not with one another [30]. In our cases, outcompeting was evident in all networks (Figs 3–5) apart from those in Rwanda. Moreover, the increasing connectivity of the lead organization was not accompanied by increasing connectivity of other innovation network stakeholders, again apart from Rwanda, indicating an increasing willingness to collaborate with the lead but not with one another. In brief, centralization occurred in all countries in terms of all network functions, but the risks of outcompeting and preference for connectivity to the lead depended on the case.

### Function-specific changes in multi-stakeholder innovation networks

The data from the Humidtropics programme in Burundi, DRC, and Rwanda indicate that the MSPs did not increase the collaboration in innovation networks (Table 6) during the period of our investigation. On the contrary, the number of organizations collaborating in the innovation networks and the connections between them decreased in all three countries (Table 5). This supports the argument that organizing MSPs does not automatically lead to more collaborative participation [6, 8, 41].

Despite the decreases in collaboration network size and number of ties in Burundi and Rwanda, knowledge exchange network size and number of ties increased (Table 5). Our data indicate that the drivers of the increase were (1) participation of new organizations in knowledge exchange (Fig 4) especially through the establishment of regional knowledge linkages with other countries in the region (Fig 4C) and (2) increasing knowledge integration of separate knowledge exchange clusters into main knowledge exchange group (Fig 4E). These data confirm that MSPs coincided with increasing expectations from several isolated organizations [24], triggering their participation. However, at the same time, all existing sub-knowledge clusters connected to the main knowledge exchange networks in Burundi and DRC in the initial data collection period disappeared (Fig 4A). Thus, it can be argued that loosely connected knowledge exchange networks with local clusters can result in competitive behaviour in the knowledge exchange network, forcing some organizations out. However, once the competitive clusters are out, innovation networks can start building higher connectivity through network closure [42]; this was visible especially in Rwanda, where no initial knowledge cluster was not connected to the MSP’s managing organization (Fig 4). These changes imply that interventions disrupt existing knowledge exchange networks and create “winners” and “losers” in terms of innovation actors’ connectivity in the areas targeted.

Change in the influence spread networks’ size and number of influence connections was case specific. Except in Burundi, influence spread network size decreased. Downward pressure on the influence spread networks attributable to the disappearance of some influence clusters (Fig 5A) was mitigated by the participation of new influential organizations (Fig 5B) and increasing influence size of the managing organization (Fig 5C). In Burundi, the number of influential participants was sufficient to substitute the decrease, but not in DRC and Rwanda.

### Common significant factors of change in multi-stakeholder innovation networks triggered by multi-stakeholder platforms

Our study showed that initial innovation network characteristics and funding provided by the MSP had significant roles in explaining the decisions of the innovation network stakeholders to continue in the networks and in explaining the difference between the continuing group of stakeholders and the stakeholders joining the innovation networks in terms of all functions (Table 6).

In our study, the number of connections at the initial survey was a significant factor explaining the changes in the innovation networks (Table 6). The likelihood of a connection between two organizations staying in the innovation networks increased significantly as the number of connections between these organizations increased in the initial period. Moreover, the number of new connections between two organizations was lower than the number of existing connections in the collaboration, knowledge exchange, and influence spread networks. In other words, in the period of our study, connections between two organizations persisted more if they were connected in multiple channels, and it took time to increase the number of connections when they were new in the innovation networks. Moreover, in our study, none of the event factors, i.e. number of events, number of specific event types, or the share of the event types, was significant, despite the variability across the countries (Table 4). Time could be a possible reason for the insignificant results, given that the effects of MSP activities involving research processes are reported to show their effects only after a time lag [32, 43–45]. In brief, our study confirms that changes triggered by MSPs happen slowly, as commonly recognized in the MSP and innovation systems literature [46–49].

The data in our study indicate that country was not a significant factor in explaining changes in innovation network functions in our cases. As the institutional context surrounding innovation networks has been shown to play a role in the effects triggered by MSPs [3, 45, 50]) insignificant country variation implies that the role of the institutional environment was reflected through other significant factors in our models: initial innovation network characteristics, funding provided, and type of activities targeted by MSPs (Table 6). Of these factors, decisions on funding and type of activities targeted by MSPs are less likely to be influenced by the specifics of the institutional environment, as in our three cases the managing organization had the dominant role in making funding and activity decisions. Thus, in our cases, initial innovation network characteristics have a high chance of sufficiently representing the effects of the institutional environment on changes triggered by MSPs.

Our data show that the likelihood of staying in all three networks increased if the organization received direct funding. Moreover, the likelihood of new collaboration, knowledge exchange, and influence connections increased significantly as the share of events funded by the MSP increased (Table 6). As limited resources cannot satisfy an increasing number of stakeholders in innovation networks [8], the fact that funding is a significant aspect implies that the number of stakeholders that can be financially incentivized is also limited. The decrease in network size and the number of connections in collaboration networks, which were relatively higher initially, combined with increasing network size and number of connections in knowledge exchange and influence spread networks, which were relatively lower in the beginning (Table 5), supports the existence of limitations introduced by funding in our cases. In addition, the data show that MSP events were highly dependent on the funding provided by MSPs (Table 6). For instance, at least two thirds of the events were fully funded by the MSPs. Dependency on funding has been reported to be high, especially in developing countries where organizations are forced to prioritize funding [35], and the number of opportunistic organizations is high in relation to the size of innovation systems [12]. In our study, all three cases are developing countries. In brief, our cases support the assertion that, in developing countries, funding dependency and opportunistic behaviour by organizations limit MSPs’ ability to affect innovation networks.

### Function-specific significant factors of change in multi-stakeholder innovation networks triggered by multi-stakeholder platforms

In terms of the decision to stay in the collaboration networks, multiple factors were significant. In addition to the initial characteristics of the innovation networks, number of initial connections, type and scale configuration of stakeholders, funding provided to organizations directly and to events, and type of activities undertaken by the MSP were all significant (Table 6). Multiple significant factors might suggest that stakeholders make their collaboration decisions based on different purposes such as accessibility to information, knowledge, and capacity development [32, 45].

Among the factors, share of events funded by the MSP has the largest effect. The likelihood of staying in innovation networks decreased dramatically as share of the events funded by the MSP increased. This confirms our previous statements on the importance of funding and dependency on funding to stay in the networks. As MSPs have limited resources, higher dependency on MSP funding for events implies less room for an organization to benefit financially from such events. When funding is important for the participating organizations, having less room for financial benefits leads to a lower likelihood of staying.

An increase in the number of types of activities decreased the likelihood of continuing and increased the likelihood of new connections in collaboration. When the first survey was administered, the priority was agronomy work through implementing activities on the ground (Table 4). It was considered that showing tangible activities would attract the interest of farmers and governments, help show progress to donors, and prevent interventions appearing to be “talking clubs”. Thus, field activities, which present activities on the ground, were operationalized, and field trials were established in many project locations. When the second survey was conducted, other goals such as improvement in nutritional status (in Burundi and DRC) and capacity building in gender issues (in Burundi) started to be implemented (Table 4). As farmer organizations are less involved with the provision of new types of activities such as nutrition and gender work, targeting nutrition and gender and implementing related activities coincided with the decreasing likelihood of farmer organizations staying in comparison to other types of innovation network stakeholders. Moreover, the relative participation of NGOs in Burundi and DRC, where they are the major providers of nutrition and gender work, increased. In brief, as the diversity of the activities increased, new stakeholders engaged in the new activities–NGOs–joined the networks, and there was a decrease in the likelihood of farmers staying in the networks, even though these had been very involved with initial activities.

Change, in terms of thematic focus, in the configuration of the innovation networks implies that thematic diversity of the objectives of the intervention is an important factor to consider in utilizing MSPs in interventions aiming to scale innovations. If the scaling of the target innovation depends on improving conditions cutting across different themes, a more intense monitoring and a more adaptive stakeholder involvement facilitation approach might be necessary in comparison to what might be required for scaling innovations that have a narrow thematic focus.

Significant factors explaining the changes in knowledge exchange and influence spread networks were fewer in number in comparison with those for collaboration networks. This confirms that collaboration networks reflect a greater diversity of participation purposes than knowledge exchange and influence spread networks. In the latter networks, in addition to the previously discussed factors (initial number of connections, funding provided to specific organizations and to events), the number of organizations in the innovation networks was initially high. As the number of organizations increased, the likelihood of organizations staying in knowledge exchange and influence spread networks decreased. Table 7 provides an overview of the changes, factors, and implications of using MSP interventions to scale innovation.

**Table 7:** Changes in innovation networks, factors influencing the changes, and the implications for scaling innovations following an R4D intervention with MSPs.

		Changes	Factors	Implications for scaling
**General**		Changes in innovation networks depend on functions	Initial network characteristics have a high influence on the changes	Influence of the intervention on scaling depends on the functional needs of the targeted innovation and the initial configuration of innovation function networks.
		Innovation networks can centralize and outcompete existing central actors	Funding is a significant factor for the changes	The interventions need to consider out-competition risk. Provision of funding is a major source of competition introduced by the intervention.
**Functions**	***Collaboration***	Extent and density of collaboration does not increase	Collaboration depends on a greater variety of factors than specific functions.	The intervention might be ineffective in scaling innovation if innovation requires extensive or intense collaboration because of the diverse nature of collaboration in innovation networks.
	***Knowledge exchange***	Extent and density of knowledge exchange might increase or decrease	1. Participation of new knowledge actors 2. Integration of small and loosely connected clusters into the main cluster 3. Funding is a significant factor 4. Type of organization is a significant factor	The intervention disrupts existing knowledge networks, creates winners and losers mostly determined by the funds provided by the intervention, and is influenced by type of stakeholder to a lesser extent. It can negatively influence scaling if there is already a knowledge cluster focused on the targeted innovation and funding of the intervention is not provided to the organizations in existing clusters.
		Existing knowledge clusters can leave the network		
	***Influence***	Extent and density of influence spread might increase or decrease	1. Participation of new influential actors 2. Funding is a significant factor 3. Influence clusters leave the network	The intervention disrupts existing influence networks, creates winners and losers mostly because of funds provided by the intervention. It can negatively influence scaling if there is already an influence cluster focused on the targeted innovation.
		Existing influence clusters can leave the network		

## Conclusions

We have confirmed that MSPs do not necessarily increase stakeholders’ participation and connectivity in innovation networks in the first few years of implementation. In addition, MSPs do not necessarily result in decentralized innovation networks. Using a participatory approach in the MSPs does not prevent centralization of innovation networks around a central actor that dominates some network functions. Although centralization does not necessarily inhibit innovation and scaling, as shown by some of our cases, it can introduce risks for innovation and scaling by crowding out some important stakeholders. Monitoring the process of change in the characteristics of innovation networks can help to identify this risk carried by MSPs.

We have shown that the influence of MSPs with the same approach to participation, connectivity, and configuration characteristics of innovations can be different. The changes in these three characteristics differ not only among the three countries studied, but also among different innovation network functions. This supports the contextual character of MSP influence on innovation networks. However, our study has also shown that there are common factors that influence the innovation network characteristics in the same manner across countries and functions, such as initial network characteristics and funding.

Initial network characteristics, especially the number of existing connections in innovation networks, were significant factors for the changes in the innovation network characteristics across all three cases. Moreover, all the innovation networks in our cases presented a high degree of continuity in many characteristics. In addition, we have shown that the influence of the case-specific institutional environment on innovation networks can be sufficiently captured by initial network characteristics. Thus, investigating innovation network characteristics using a network approach in the early phases of MSPs can contribute to MSP performance in improving innovation and scaling by capturing the effect of contextual characteristics and identifying target organizations and connections among innovation networks. Financial incentivizing of organizations, either directly or indirectly through events, can be an effective tool for MSPs to influence the change in innovation networks towards better innovation and scaling.

We should, however, acknowledge that, although the MSPs studied used the same approach and were managed by the same organization, heterogeneities can occur, as commonly observed in complex interventions. Further exploration of the heterogeneities of MSPs could improve our study’s conclusions. We also anticipate a difference in the speed of change in innovation networks in different countries and for different functions. As our data did not capture a long period and time was a factor in the changes in the innovation networks, a better understanding of the phases of the innovation networks can shed further light on changes triggered by an MSP in innovation networks.

## Supporting information

S1 File Questionnaire English t = 1 survey. (DOCX)

S2 File Questionnaire English t = 2 survey. (DOCX)

S3 File Questionnaire French t = 1 enquête. (DOCX)

S4 File Questionnaire French t = 2 enquête. (DOCX)
