# Supplementary material for: Effects of multi-stakeholder platforms on multi-stakeholder innovation networks: Implications for research for development interventions targeting innovations at scale
Source: PLoS One. 2018 Jun 5;13(6):e0197993. doi: 10.1371/journal.pone.0197993 (PMC5988278; doi:10.1371/journal.pone.0197993)
Supplement: S2 File — (DOCX) [file pone.0197993.s002.docx]

Name: _________________________________________________

Gender: Male/ Female

Age: _________________________________________________

Organisations you represent:

_________________________________________________

_________________________________________________

_________________________________________________

_________________________________________________

**List all organisations you collaborate with:**

__________________________________________________________

__________________________________________________________

__________________________________________________________

__________________________________________________________

__________________________________________________________

__________________________________________________________

__________________________________________________________

__________________________________________________________

___________________________________________________________

__________________________________________________________

__________________________________________________________

__________________________________________________________

__________________________________________________________

__________________________________________________________

__________________________________________________________

__________________________________________________________

__________________________________________________________

__________________________________________________________

__________________________________________________________

__________________________________________________________

__________________________________________________________

__________________________________________________________

__________________________________________________________

__________________________________________________________

**Additional questions :**

1. In the last year, the R4D platform has enforced collaboration between your organization and other partners (please circle appropriate) :

- I strongly disagree
- I disagree
- Neutral
- I agree
- I strongly agree

1. In the last year, the R4D platform has enforced exchangeof knowledge between your organization and other partners (please circle appropriate answer) :

- I strongly disagree
- I disagree
- Neutral
- I agree
- I strongly agree

1. In the last year, the R4D platform has allowed your organization and others to influence policy makers (please circle appropriate answer) :

- I strongly disagree
- I disagree
- Neutral
- I agree
- I strongly agree

1. In your opinion, what is necessary for the R4D platform to improve in terms of renforcement of collaboration, exchange of information and influence on policy makers ?

| Write your answer here : |
| --- |

| Continue here: |
| --- |

6. Which type of organizations are more effective in improving capacity of innovation in the agricultural and food sectors? Please choose maximum 2 for each group

| Farmer Organizations |  |
| --- | --- |
| NGO or CSO |  |
| Business |  |
| Researchers or universities |  |
| Government |  |
| Others ( |  |

7. Which linkages are more effective in scaling innovations in the agricultural and food

|  | Local | Provincial | National | Supranational |
| --- | --- | --- | --- | --- |
| Local (village, district) |  |  |  |  |
| Provincial |  |  |  |  |
| National |  |  |  |  |
| Supranational |  |  |  |  |

sectors? Please mark the 3 most important boxes. The linkages can be both between different groups and the same groups.

8. Which linkages are more effective in scaling innovations in the agricultural and food sectors? Please mark the most important box.

| Local (village, district) |  |
| --- | --- |
| Provincial |  |
| National |  |
| Supranational |  |

|  | Farmer | NGO/CSO | Business | Academy | Government |
| --- | --- | --- | --- | --- | --- |
| Farmer Organizations (Farmer) |  |  |  |  |  |
| NGO or CSO |  |  |  |  |  |
| Business |  |  |  |  |  |
| Academy (Researchers or universities) |  |  |  |  |  |
| Government | s |  |  |  |  |
