# Supplementary material for: Effects of multi-stakeholder platforms on multi-stakeholder innovation networks: Implications for research for development interventions targeting innovations at scale
Source: PLoS One. 2018 Jun 5;13(6):e0197993. doi: 10.1371/journal.pone.0197993 (PMC5988278; doi:10.1371/journal.pone.0197993)
Supplement: S3 File — (DOCX) [file pone.0197993.s003.docx]

Nom: _________________________________________________

Genre: Homme/ Femme

Age: _________________________________________________

Organisations que vous représentez:

_________________________________________________

_________________________________________________

_________________________________________________

_________________________________________________

**Liste toutes les organisations avec vous collaborez:**

__________________________________________________________

__________________________________________________________

__________________________________________________________

__________________________________________________________

__________________________________________________________

__________________________________________________________

__________________________________________________________

__________________________________________________________

__________________________________________________________

__________________________________________________________

__________________________________________________________

__________________________________________________________

__________________________________________________________

__________________________________________________________

__________________________________________________________

__________________________________________________________

__________________________________________________________

__________________________________________________________

__________________________________________________________

__________________________________________________________

__________________________________________________________

__________________________________________________________

__________________________________________________________

__________________________________________________________

__________________________________________________________

__________________________________________________________

__________________________________________________________

__________________________________________________________

__________________________________________________________

__________________________________________________________

__________________________________________________________

__________________________________________________________

__________________________________________________________

__________________________________________________________

__________________________________________________________

__________________________________________________________

__________________________________________________________

__________________________________________________________

__________________________________________________________

__________________________________________________________

__________________________________________________________
