# Supplementary material for: Effects of multi-stakeholder platforms on multi-stakeholder innovation networks: Implications for research for development interventions targeting innovations at scale
Source: PLoS One. 2018 Jun 5;13(6):e0197993. doi: 10.1371/journal.pone.0197993 (PMC5988278; doi:10.1371/journal.pone.0197993)
Supplement: S4 File — (DOCX) [file pone.0197993.s004.docx]

Nom: _________________________________________________

Genre: Homme/ Femme

Age: _________________________________________________

Organisations que vous représentez:

_________________________________________________

_________________________________________________

_________________________________________________

_________________________________________________

**Liste toutes les organisations avec vous collaborez:**

__________________________________________________________

__________________________________________________________

__________________________________________________________

__________________________________________________________

__________________________________________________________

__________________________________________________________

__________________________________________________________

__________________________________________________________

__________________________________________________________

__________________________________________________________

__________________________________________________________

__________________________________________________________

__________________________________________________________

__________________________________________________________

__________________________________________________________

__________________________________________________________

__________________________________________________________

__________________________________________________________

__________________________________________________________

__________________________________________________________

__________________________________________________________

__________________________________________________________

__________________________________________________________

__________________________________________________________

__________________________________________________________

__________________________________________________________

__________________________________________________________

__________________________________________________________

__________________________________________________________

__________________________________________________________

__________________________________________________________

__________________________________________________________

__________________________________________________________

__________________________________________________________

__________________________________________________________

__________________________________________________________

__________________________________________________________

__________________________________________________________

__________________________________________________________

__________________________________________________________

__________________________________________________________

**Questions additionnelles :**

1. Au cours de l’année dernière, la plate forme R4D a renforce la collaboration entre votre organisation et les autres partenaires (svp encerclez votre réponse plus appropriée) :

- Je désapprouve complètement
- Je désapprouve partiellement
- Je suis neutre
- J’approuve partiellement
- J’approuve complètement

1. Au cours de l’année dernière, la plate forme R4D a renforce l’échange de connaissances entre votre organisation et les autres partenaires (svp encerclez votre réponse plus appropriée) :

- Je désapprouve complètement
- Je désapprouve partiellement
- Je suis neutre
- J’approuve partiellement
- J’approuve complètement

1. Au cours de l’année dernière, la plate forme R4D a permis à votre organisation et les autres d’influencer les décideurs politiques (svp encerclez votre réponse plus appropriée) :
   - Je désapprouve complètement
   - Je désapprouve partiellement
   - Je suis neutre
   - J’approuve partiellement
   - J’approuve complètement
2. Qu’est ce qui est nécessaire pour améliorer le fonctionnement de la plate forme R4D en termes de renforcement de la collaboration, échange d’information et l’influence sur les décideurs politiques ?

| Votre réponse question 5 : |
| --- |

6. Which type of organizations are more effective in improving capacity of innovation in the agricultural and food sectors? Please choose maximum 2 for each group

| Farmer Organizations |  |
| --- | --- |
| NGO or CSO |  |
| Business |  |
| Researchers or universities |  |
| Government |  |
| Others ( |  |

7. Which linkages are more effective in scaling innovations in the agricultural and food sectors? Please mark the 3 most important boxes. The linkages can be both between different groups and the same groups.

| Local (village, district) |  |
| --- | --- |
| Provincial |  |
| National |  |
| Supranational |  |

|  | Local | Provincial | National | Supranational |
| --- | --- | --- | --- | --- |
| Local (village, district) |  |  |  |  |
| Provincial |  |  |  |  |
| National |  |  |  |  |
| Supranational |  |  |  |  |

|  | Farmer | NGO/CSO | Business | Academy | Government |
| --- | --- | --- | --- | --- | --- |
| Farmer Organizations (Farmer) |  |  |  |  |  |
| NGO or CSO |  |  |  |  |  |
| Business |  |  |  |  |  |
| Academy (Researchers or universities) |  |  |  |  |  |
| Government |  |  |  |  |  |

8. Which linkages are more effective in scaling innovations in the agricultural and food sectors? Please mark the most important box.
